# Canine Visceral Leishmaniasis: A Histological and Immunohistochemical Study of Fibropoiesis in Chronic Interstitial Pneumonitis

**DOI:** 10.3390/microorganisms12050941

**Published:** 2024-05-07

**Authors:** Frederico C. Gonçalves, Ramon de Alencar Pereira, Adriano Francisco Alves, Aldair Pinto Woyames Junio, Ricardo T. Fujiwara, David M. Mosser, Helida Monteiro Andrade, Geovanni D. Cassali, Enio Ferreira, Wagner Luiz Tafuri

**Affiliations:** 1 Departmento de Patologia, Instituto de Ciências Biológicas, Universidade Federal de Minas Gerais, Campus Pampulha, Av. Antônio Carlos, Belo Horizonte 6627, Brazilramon2alencar@gmail.com (R.d.A.P.); adrianofalves@gmail.com (A.F.A.); aldairwpinto@gmail.com (A.P.W.J.); geovanni.cassali@gmail.com (G.D.C.); eniofvet@hotmail.com (E.F.); 2 Departmento de Parasitologia, Instituto de Ciências Biológicas, Universidade Federal de Minas Gerais, Campus Pampulha, Av. Antônio Carlos, Belo Horizonte 6627, Brazil; fujiwara@icb.ufmg.br (R.T.F.); helidandrade@gmail.com (H.M.A.); 3 Department of Cell Biology and Molecular Genetics, Maryland Pathogen Research Institute, University of Maryland, College Park, MD 20742, USA; dmosser@umd.edu

**Keywords:** canine visceral leishmaniasis, chronic interstitial pneumonitis, fibrosis

## Abstract

We studied some fibrotic aspects of chronic interstitial pneumonitis in the lungs of dogs infected with *Leishmania infantum*. The lungs of eleven naturally infected dogs, twelve experimentally infected with two distinct strains of *L. infantum* (BH401 and BH46), and six uninfected (controls) dogs, were analyzed by histological, parasitological, and immunohistochemical studies. Conventional histology (HE), collagen deposition (Gomori’s silver staining for reticulin collagen fibers), and immunohistochemistry for myofibroblast characterization were carried out based on the cellular expression of alpha-smooth muscle actin, vimentin, cytokeratin, E-cadherin, snail antigen homologue 1 (SNAI1) (Snail), and the cytokine expression of transforming growth factor-beta (TGF-β). Parasitological screening was carried out using conventional polymerase chain reaction (PCR) and the immunohistochemical reaction of streptavidin–peroxidase for visualizing *Leishmania* amastigotes. Dogs naturally infected with *L. infantum* and experimentally infected with *L. infantum* BH401 strains showed intense interstitial pneumonitis characterized by thickening of the alveolar septa as a consequence of an intense diffuse and focal (plaques) chronic exudate of mononuclear cells associated with fibrogenesis. The expression of alpha-actin, vimentin, and TGF-β was higher in the lung interstitium of all infected dogs than in the other two groups (BH46 strain and controls). Moreover, in both the naturally and experimentally infected dog (BH401 strain) groups, the expression of Snail was moderate to intense in contrast to the other groups. Based on these immunohistochemical results, we concluded that mesenchymal cells are active in promoting changes in the extracellular matrix in the lungs of dogs naturally and experimentally infected with *L. infantum*, but it depends on the virulence of the parasite.

## 1. Introduction

Respiratory clinical symptoms such as a dry and persistent cough are reported in human visceral leishmaniasis (HVL), but most often, these symptoms are considered to result from bacterial bronchopneumonia or viral pneumonia. Currently, this type of cough is known to be caused by interstitial pneumonitis (a chronic interalveolar inflammatory response), but there are few studies in the literature despite the fact that this pulmonary pathology is frequent in HVL [1,2] and in canine visceral leishmaniasis (CVL) [3,4]. In both diseases, the first descriptions of interstitial pneumonitis were based on the presence of parasites in the lungs of human beings [5] and dogs [6]. Following this, it seems that, in HVL, the first histological studies describing interstitial pneumonitis were performed around 1960 [7,8], whereas for CVL, these morphological works come from reports of dogs naturally infected with *Leishmania (Leishmania) donovani* around 1976 [9,10].

Interstitial pneumonitis, or interstitial lung disease, comprises a broad range of conditions, including diffuse and focal inflammatory disorders that lead to the thickening of the alveolar or interlobular septa that make up the lung interstitium. This thickening is characterized by changes in the epithelial and endothelial cells in the alveolar walls, serofibrinous exudation into the alveoli, and hypercellularity of inflammatory cells, along with extracellular matrix (ECM) changes leading to fibrosis of the alveolar septa (Duarte et al., 1989 [2]). However, studies of the pathogenesis of chronic interstitial pneumonia associated with fibrosis in CVL are scarce. The lung is less often parasitized and thus not the focal point of the infection in dogs [3,4].

As already reported in the literature, intense fibrosis occurs in the lungs of dogs naturally infected with *Leishmania (Leishmania) infantum*, independent of the lung tissue parasite load or clinical status of the animals [4,11]. Thus, the aim of this work was to characterize the expression of cell markers related to fibrosis in chronic interstitial pneumonitis in dogs naturally and experimentally infected with *L. infantum*. For the experimental protocols, we used two distinct strains of *L. infantum* (BH401 and BH46, described later). We employed alpha-actin (α-SMA), vimentin, and Snail antigen homologue 1 (SNAH 1—Snail) to assess the presence of mesenchymal cells (myofibroblasts). The transforming-growth-factor-beta (TGF-β1) cytokine was also targeted, as it is a key cytokine in the development of fibrosis. Cytokeratin and E-cadherin expression for epithelial cell characterization was carried out in parallel with TGF-β1 due to its ability to differentiate and sustain epithelial–mesenchymal transition (EMT) activity and increase the survival of mesenchymal cells.

## 2. Materials and Methods

### 2.1. Samples and Animals

Paraffin-embedded tissue samples belonging to the collection of the Laboratory of *Leishmaniasis* (Departamento de Patologia Geral, Instituto de Ciências Biológicas (ICB), Universidade Federal de Minas Gerais (UFMG) were selected for this study. Twenty-nine paraffin blocks were distributed as follows:

Group of dogs naturally infected with *L. infantum*: Eleven dogs naturally infected with *L. infantum*, all adult males, mixed-breeds with 8-to-15 kg body mass, were obtained from the Zoonosis Center of Belo Horizonte (Municipality of Ribeirão das Neves, Minas Gerais State, Brazil). All had a record of positive serological tests for *Leishmania* diagnosis, such as indirect immunofluorescence and ELISA [12]. Parasitological tests, including bone marrow smears, confirmed the infection.

Group of dogs experimentally infected with *L. infantum*: Twelve male beagles purchased from dog breeders in regions of Brazil nonendemic for *Leishmania* (Paraná State, South Brazil) were used. All were vaccinated against leptospirosis, distemper, coronavirus, canine adenovirus type 2, infectious hepatitis, para-influenza, and parvovirus. The beagles, five-to-six months old, were divided into two subgroups. Group BH401, composed of five animals, was infected with 5 × 10^7^/mL i.v. promastigote forms of *L. infantum* strain *MCAN/BR/2002/BH401*; group BH46 was composed of seven animals infected with 5 × 10^7^/mL i.v. of promastigotes forms of *L. infantum strain MHOM/BR/1972/BH46*. Both parasite strains were inoculated at the end of the logarithmic growth phase after culture in α-MEM culture medium (GIBCO BRL) supplemented with 10% inactivated fetal calf serum (Sigma-Aldrish, Darmstadt, Germany), 200 U penicillin/L (Sigma), and 100 µg/mL streptomycin (Sigma), pH 7.4, at 24–26 °C [13].

At ICB/UFMG, the experimentally infected dogs and control dogs were maintained for more than eighteen months (almost two years) under healthy conditions to avoid contamination with *Leishmania*, in accordance with the Guiding Principles for Biomedical Research. The ICB kennels were treated with pyrethroid insecticide, and their entire wall lengths were protected with a stainless-steel screen. The experiments were monitored by veterinarians, and invasive procedures were performed following the standards of ethical procedures for trial animals and biosecurity. All dogs were tested monthly after infection for the presence of *Leishmania* antibodies by indirect immunofluorescence and ELISA [12]. *L. infantum* infection was also confirmed by real-time polymerase chain reaction (RT-PCR) in samples of tissues, such as livers, spleens, or cervical lymph nodes, as previously described [13,14].

Group of uninfected animals (controls): Six uninfected beagle dogs were used as controls. Parasitological and serological tests were performed to confirm the absence of infection for *Leishmania*.

All groups of dogs had negative serological tests (before euthanasia) for *Erlichia*, *Mycoplasma*, and *Babesia* to assess for the possibility of coinfection [14].

### 2.2. Ethics Statement

This study was approved by the Comissão de Ética no Uso de Animais (CEUA) UFMG (Protocols 198/2014 and 317/2015).

### 2.3. Histology and Morphometric Analysis of Collagen Deposition

All lung samples embedded in paraffin were processed for histology with 3 µm thick sections: (a) routine hematoxylin–eosin (H&E) staining, (b) Gomori’s ammoniacal silver staining for collagen fibers (reticular fibers), (c) Grocott staining for differential fungi and yeast identification, and (d) Good Pasture staining for the presence of bacteria (Gonçalves et al., 2003 [4]). For collagen studies, all lung fragments were stained with Gomori’s ammoniacal silver staining, where the fibrillar collagen fibers are easily observed in black. Thus, a histomorphometrical analysis was carried out in order to characterize the pulmonary interstitial collagen deposition. This analysis was performed using an Axiolab light microscope (Zeiss, Oberkochen, Germany) with ×440 resolution. The images were transferred to a computer video screen, using software, and relayed to a computer-assisted image analysis system (Kontron Elektronic/Carl Zeiss, Oberkochen, Germany). Using a digital pad, the total area occupied by stained collagen fibers was measured from real images and segmented to generate binary images. The results are expressed in square micrometers (Kontron Elektronic/Carl Zeiss, Germany) [4,5,6,7,8,9,10,11,12,13,14].

### 2.4. Immunohistochemistry Assays

Immunohistochemical assays were carried out on paraffin-embedded lung tissue samples for markers of epithelial cells, such as cytokeratin and E-cadherin. In addition, mesenchymal cells were characterized by the expression of alpha-smooth muscle actin (α-SMA), vimentin, and Snail. An immunohistochemical assay for TGF-β1 was also conducted. The protocols followed those of Madeira et al. (2016) [15], who investigated fibropoiesis mechanisms. The lung sections were dewaxed, hydrated, and immersed in 4% hydrogen peroxide (30 *v*/*v*) in 0.01 M phosphate-buffered saline (PBS; pH 7.2) for 20 min to block endogenous peroxidase activity. The slides were then rinsed and submerged in diluted goat serum (1:100 dilution) to block nonspecific immunoglobulin absorption. We carried out immunohistochemical protocols specific to each antigen. For α-SMA, vimentin, cytokeratin, E-cadherin, Snail, and TGF-β antigen characterization, we employed specific primary antibodies (Table 1). The dewaxed and hydrated lung tissue sections were treated with antigen retrieval solution (1% Target Retrieval Solution-Dako, Carpinteria, CA, USA) for 30 min in a water bath at 98 °C. The slides were treated with methanol containing 0.3% hydrogen peroxide for 15 min at room temperature to inactivate endogenous peroxidase. The assay for TGF-β protein was similar, but the slides were washed with 0.01 M PBS and 1% Tween-20. Antigen retrieval was conducted using a Pascal pressure cooker (DakoCytomation, Carpinteria CA, USA) according to the manufacturer’s instructions.

For the detection of amastigotes of *Leishmania* in paraffin-embedded tissues, serum obtained from a dog naturally infected with *Leishmania* (*L.*) *infantum* (a dog from the metropolitan area of Belo Horizonte, MG, Brazil) was diluted 1:100 with 0.01 M PBS and used as the primary antibody as original descrbed [16]. The slides were incubated in humid conditions at 4 °C for 18–22 h, washed in PBS, and incubated with biotinylated goat anti-mouse and anti-rabbit immunoglobulin (Dako, Carpinteria, CA, USA; LSAB2 kit). Finally, the slides were washed and incubated with streptavidin–peroxidase complex (Dako; LSAB2 kit) for 20 min at room temperature. The reactions were visualized by applying a Liquid DAB + Substrate Chromogen System (Dako, K3468) for 5 to 10 min, with H&E counterstaining for 5 min.

### 2.5. Semiquantitative Immunohistochemistry Analysis

Immunolabeled cells positive for α-SMA, vimentin, snail, and TGF-β stained brown were quantified in each tissue sample. Twenty fields of each slide of lung tissue were analyzed microscopically with a 40× objective and scored as minor (+) (<20% of each field), moderate (++) (20–50% of each field), or intense (+++) (>50% of each field) [15].

### 2.6. Molecular Techniques—Polymerase Chain Reaction (PCR)

#### 2.6.1. Extraction of DNA Material

We investigated the presence of *Leishmania* in the lungs of all animals naturally and experimentally infected with *L. infantum* (BH46) only by conventional PCR because we did not find amastigotes in the tissue by optical microscopy, including immunohistochemistry. DNA from formalin-fixed paraffin-embedded tissue was purified using a NucleoSpin Tissue extraction kit (Macherey-Nagel, Düren, Germany). Up to 25 mg of tissue was obtained using a sterile razor blade (Chrome platinum, BIC), and the paraffin was carefully removed by three cycles of immersion in xylene for 5 min each, followed by subsequent immersion in absolute ethanol. The samples were dried at room temperature to remove the excess ethanol and subjected to lysis and DNA extraction steps according to the kit protocol described by da Silva et al. (2012) [17].

#### 2.6.2. DNA Amplification

Conventional PCR was also carried using the specific primers used were a sense, 5′-CTGGATCATTTTCCGATG-3′; and an antisense, 5′-TGATACCACTTATCGCACTT-3′, which amplify a fragment of ~350 base pairs of the ribosomal internal transcribed spacer. The reaction was performed by adding 2 μL of genomic DNA to a mixture containing 10 μL buffer (GoTaq Green MasterMix, Promega, Madison, WI, USA), 10 pmol of each primer, and 6 μL nuclease-free water to a final volume of 20 μL. The reaction controls were DNA-extracted from cultured *Leishmania infantum* promastigotes (MHOM/BR/1967/BH46) and DNA-extracted from the lung tissue of dogs known to be infected or negative for *Leishmania* by serological methods. To confirm the integrity of the DNA, PCR targeting a dog housekeeping gene (GAPDH, sense: 5′-TTCCACGGCACAGTCAAG3′, antisense: 5′-ACTCAGCACCAGCATCAC-3′) was conducted under the same conditions. The reaction was carried out in a conventional thermal cycler (PTC-100, MJ Research Inc., Waltham, MA, USA) with initial denaturation at 95 °C for 40 s, followed by 35 cycles of annealing at 53 °C for 40 s, extension at 72 °C for 50 s, and denaturation at 95 °C for 40 s followed by a final extension at 72 °C for 5 min. The PCR-amplified products were analyzed by electrophoresis in a 6% polyacrylamide gel, and the amplified fragments were visualized by conventional silver staining [17].

### 2.7. Statistical Analysis

Histological results obtained in a fully randomized design were log-transformed, and the means for each group were compared by GraphPad Prisma 7 software. The One-Way ANOVA test was used to analyze the parametric data between the groups, and the Kruskal–Wallis test was used for the non-parametric data; in both cases, the multiple comparison post-test between the columns was applied. The *p* values < 0.0001 were considered significant for all tests.

## 3. Results

### 3.1. Chronic Interstitial Pneumonitis—Hematoxylin–Eosin (HE) Histological Analysis

**Figure 1:**
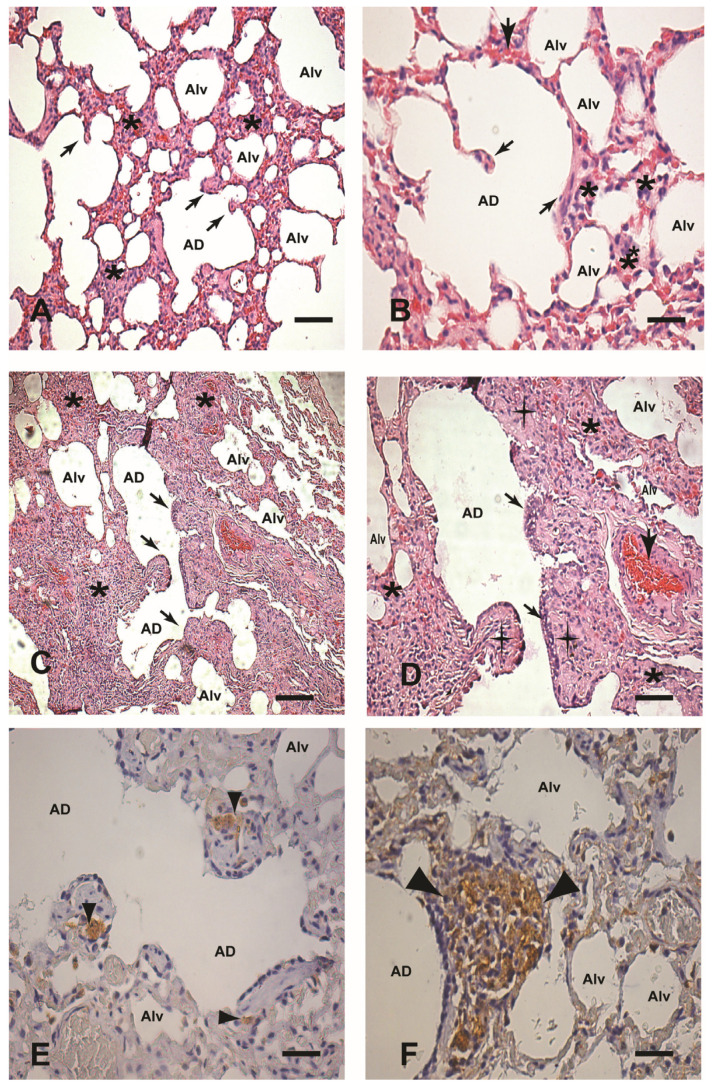
(**A**–**F**) Paraffin-embedded lung sections from uninfected dogs (controls) and dogs naturally and experimentally infected with *L. infantum* (BH401 strain). (**A**,**B**) Control dog: (**A**) Lower magnification showing pulmonary interstitial cells (alveolar wall) (asterisks), 220×. Note the regular pulmonary knob structures (black arrows). (**B**) Higher magnification showing interstitial cells into the interalveolar septum (asterisks), 440×. (**C**,**D**) Naturally infected dog: (**C**) Lower magnification showing a conspicuous thickness in the interalveolar septum (asterisks) associated with chronic interstitial pneumonitis in comparison to controls, 220×. (**D**) Presence of an intense chronic cell exudate into the intra-alveolar septum (asterisks); note the thickness of the pulmonary knob structures (black cross), the hypertrophy and hyperplasia of the pneumocytes (black arrows), and the hyperemia (large black arrow), 440×. (**E**,**F**) Experimentally infected dog (BH406 strain): Note immunolabeled amastigote forms of *Leishmania* (in brown) localized in the pulmonary interstitium (arrowheads), 440×. In (**F**), note the numerous macrophages loaded with immunolabeled parasites in the granuloma formation (large arrowheads), 440×. (**A**–**D**) Hematoxylin and eosin staining. (**E**,**F**) Immunohistochemistry streptavidin–peroxidase method with counterstaining with Harris hematoxylin. Alveolar duct (AD). Alveolus (Alv). Bars (**A**,**C**) = 20 µm; Bars (**B**,**D**–**F**) = 60 µm.

In general, intense chronic interstitial pneumonitis was the main lesion observed, but it was only notable in naturally infected dogs and dogs experimentally infected with the BH401 strain. Indeed, this chronic pathology was minor or absent in the lung tissue sections from dogs experimentally infected with the BH46 strain and in controls. Under microscopic analysis, chronic interstitial pneumonitis was characterized by thickening of the alveolar wall (alveolar septa) as a result of mononuclear cell infiltrates consisting of macrophages, plasma cells, lymphocytes, rare neutrophils, and an occasional eosinophil. This cellular exudate was generally mainly diffuse throughout, mainly into the alveolar septa, but in some cases, mononuclear cells also appeared in patches. In addition, blood vessel congestion (alveolar capillaries) was notably hyperemic. However, there was no alveolar damage or alveolar capillary alterations (except hyperemia) and/or oedema or intra-alveolar inflammatory cells. A chronic inflammatory exudate around the bronchioles was also observed in parallel to the interstitial pneumonitis, but only in the naturally and BH401 experimentally infected dogs (Figure 1A–D). Also, in pulmonary interstitium, under optical microscopy (immunohistochemistry), we found intracellular amastigote forms of *Leishmania* associated with the inflammatory exudate in all dogs experimentally infected with *L. infantum* strain BH401 and seven naturally infected dogs (Figure 1E,F). However, we did not find any amastigotes of *Leishmania* in the lung tissue of dogs experimentally infected with the *L. infantum* BH46 strain and controls. Thus, we applied PCR assays in these cases. PCR was successful (positive for *Leishmania* DNA) in four of seven dogs of this group. Otherwise, we found positive results of three more cases out of eleven dogs naturally infected with *L. infantum.* All controls were PCR-negative. It is important to say that all infected dogs showed parasites in other organs, such as the liver, spleen, and cervical lymph nodes [17].

### 3.2. Chronic Interstitial Pneumonitis—Fibropoiesis Histochemical Analysis

In addition to the presence of a chronic cellular exudate in the interalveolar septa as described, we also found a peculiar morphological alteration of the terminal respiratory bronchioles, alveolar ducts, and alveoli walls: hypertrophy and hyperplasia of pneumocytes. In some cases, the epithelium consisted of four or five layers of cuboid nonciliated pneumocytes in alveolar ducts and two or three layers in alveolar septa. Moreover, all infected dogs showed a remarkable histological alteration of the alveolar ducts. We already know that alveolar ducts are formed by knobs (alveolar pulmonary structures) lined by simple cuboidal epithelium, without cilia or club cells. The epithelium overlies very thin layers of connective tissue and strands of smooth muscle. In all infected dogs, except for the BH46 group, hypertrophy and hyperplasia of these knobs, followed by the presence of interstitial chronic cell exudate, were remarkable. Otherwise, many of these “reactive knobs” projected into the lumen of the alveoli, alveolar ducts, and terminal bronchioles.

**Figure 2:**
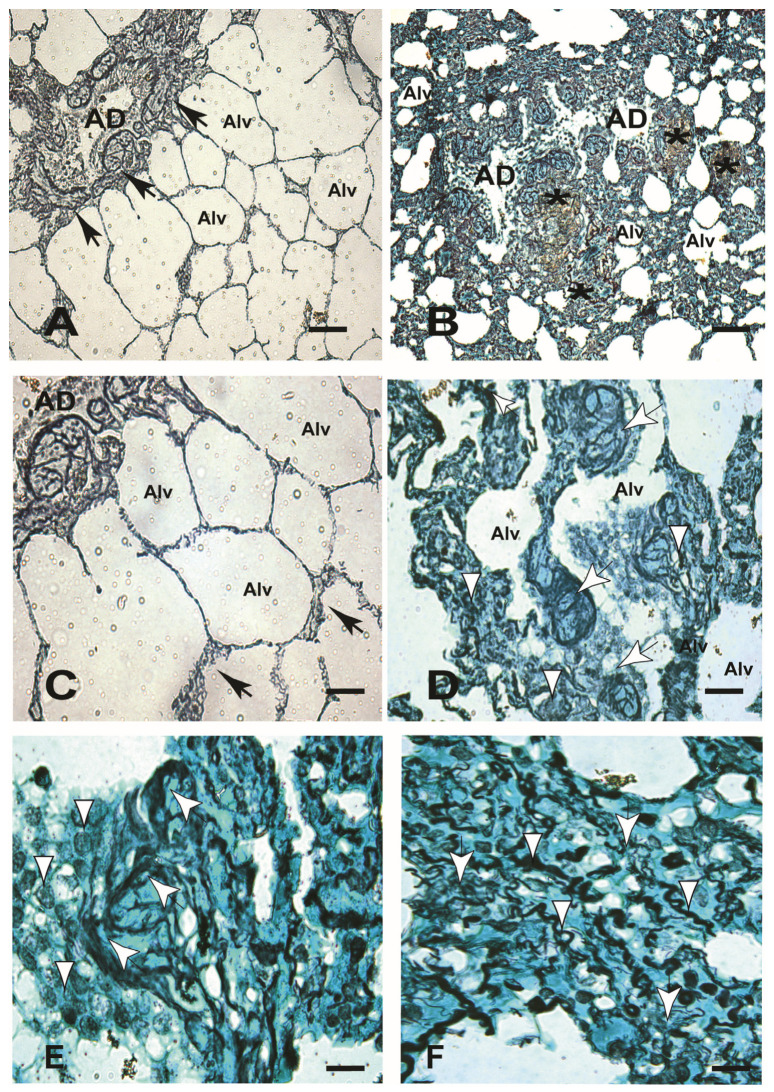
(**A**–**F**) Paraffin-embedded lung sections from an uninfected dog (control) and an experimentally infected dog with *L. infantum* (BH 401 strain). (**A**,**C**) Control dog: (**A**) Lower magnification showing a delicate distribution of reticular positive collagen fibers in black mainly observed in alveolar ducts (black arrows), 220×. (**C**) Higher magnification confirming a discrete presence of reticular collagen fibers in the intra-alveolar septa (black arrows), 440×. (**B**–**F**) Experimentally infected dog (BH401 strain). In (**B**), note a conspicuous chronic interstitial pneumonitis characterized by the thickening of the alveolar wall (alveolar septa) because of mononuclear cell exudate and collagen deposition (fibrosis) in comparison to the control (asterisks), 220×. (**D**) Higher magnification showing increased silver staining of collagen fibers in the alveolar septa (white arrowheads) and throughout the alveolar wall, mainly notable in knobs (structures of the lung parenchyma), where many of them project into the lumen of the alveolus or alveolar duct (white arrows), 440×. (**E**) Note a conspicuous black reticular fibers deposition in the alveolar septa as dense tangled structures described as “balls of black wool” (white arrows). Also, observe the epithelium hypertrophy and hyperplasia of the alveolar wall (white arrowheads), 440×. (**F**) Deep black reticular fibers’ deposition in the lung interstitium, sometimes thin (white arrows) and/or dense (arrowheads), can be observed. However, these black reticular fibers are arranged in various directions and often in a spiral or “stitch-like shape of the shortened and narrowed type” (arrowheads); 440×. Gomori’s ammoniacal silver staining. Alveolar Duct (AD). Alveolus (Alv). Bars (**A**,**B**) = 20 µm. Bars (**C**–**F**) = 60 µm.

Thus, we decided to investigate possible collagen deposition in the interstitial space (pulmonary interstitium) by using Gomori’s ammoniacal silver staining. In fact, this histological staining revealed numerous reticular fibers (collagen III) that were stained black immediately beneath the epithelium of the terminal respiratory bronchioles and the alveolar ducts and within the alveolar septa. Smooth musculature layers were interrupted by masses of reticular fibers forming dense tangled structures, previously described by Gonçalves et al. (2003) [4] as “balls of black wool” (Figure 2). Fibrosis was confirmed by a morphometric quantification analysis of the collagen fibers (interalveolar septa), which revealed higher collagen deposition in the lungs of naturally infected dogs and experimentally infected dogs infected with the BH401 strain than in the BH46 strain and control groups.

Sections stained with Grocott and Good Pasture were negative for fungi and bacteria, respectively, in all groups, including the controls.

### 3.3. Chronic Interstitial Pneumonitis—Fibropoiesis Immunohistochemical Analysis

**Figure 3:**
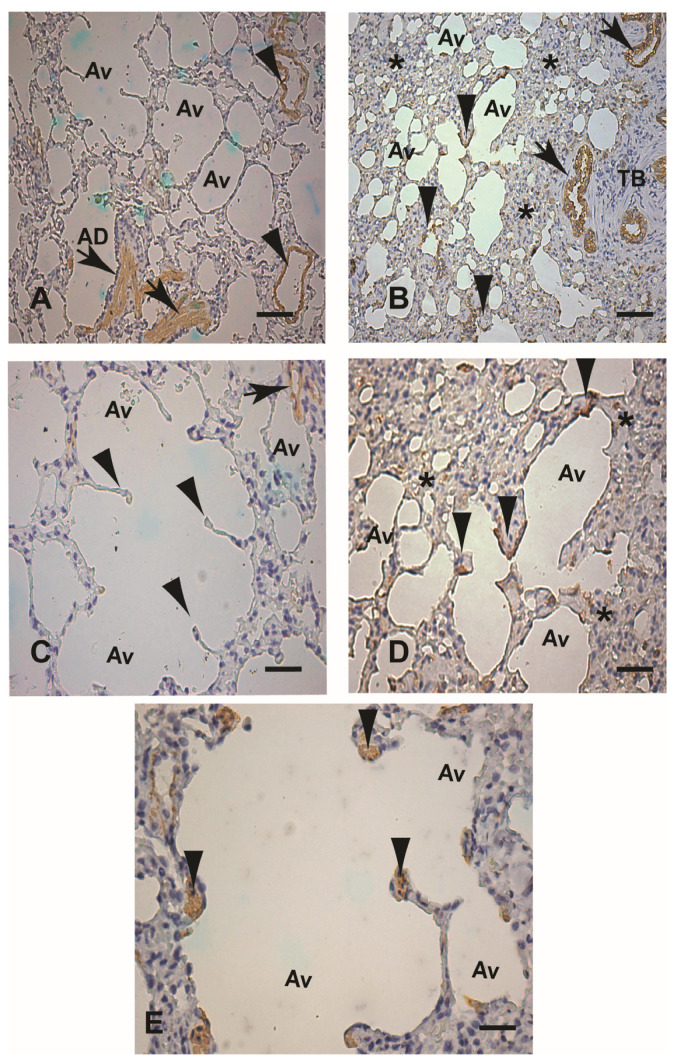
(**A**–**E**) Alfa actin (α-SMA) immunohistochemical expression in paraffin-embedded lung tissue samples from an uninfected dog (control) and an experimental dog infected with *Leishmania infantum* (BH401 strain). (**A**,**C**) Control dog: (**A**) Lower magnification showing restricted α-SMA-positive cell expression in cells compatible with smooth muscle fibers of alveolar duct walls (black arrows) and arteriole walls (black arrowheads), 220×. (**C**) Higher magnification shown in the upper right corner of the figure, α-SMA positivity only in the arteriolar wall (black arrow). Note the alveolar knobs and pulmonary structures without any positive staining (arrowheads), 440×. (**B**,**E**) Experimentally infected dog (BH401 strain): (**B**) Lower magnification to verify α-SMA positive cells in the alveolar hyperplastic epithelium wall and in the interstitium, just beneath the epithelium comprising the hyperplastic and hypertrophy knobs (black arrows). Note the intense chronic interstitial pneumonitis where the α-SMA positive cells are located (black asterisks), 220×. (**D**) Higher magnification showing the hyperplastic and hypertrophy knob structures of the alveolar ducts and alveoli (black arrowheads) and positive interstitial cells (black asterisks), 400×. (**E**) Note the positive α-SMA expression staining pattern just underneath the alveolar wall epithelium (knob pulmonary structures—black arrowheads), 440×. Immunohistochemistry streptavidin–peroxidase method with counterstaining with Harris hematoxylin. Alveolar duct (AD). Alveolus (Av). Terminal bronchiole (TB). Bars (**A**,**B**) = 20 µm. Bars (**C**–**E**) = 60 µm.

**Scheme 1:**
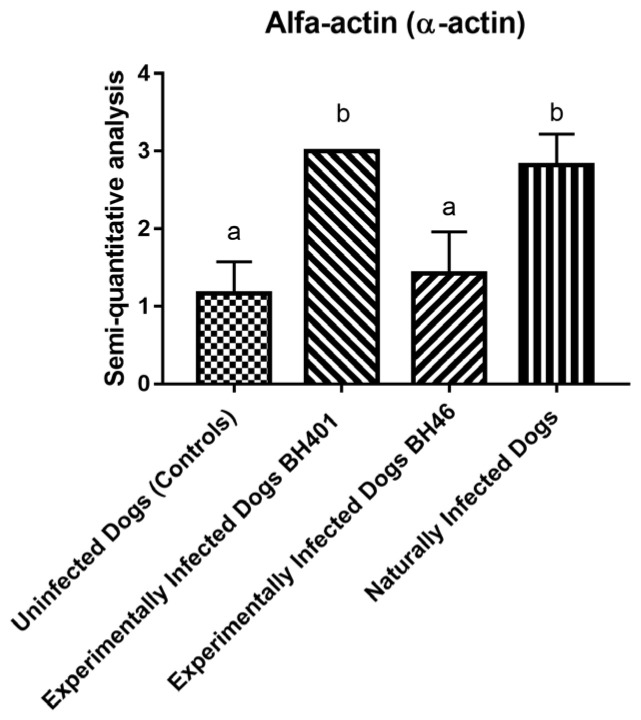
α-SMA cell expression morphometric analysis (semiquantitative study) among dog groups: dogs naturally infected with *L. infantum*, experimentally infected dogs with *Leishmania infantum* BH 401 strain, experimentally infected dogs with *Leishmania infantum* BH 46 strain, and uninfected dogs (controls). The “a” and “b” denote statistically significant difference (*p* < 0.0001).

α-SMA-positive cells were observed throughout the lung parenchyma of all dogs, but primarily in cells compatible with the smooth muscle fibers of blood vessel walls (arterioles), bronchiole walls, and terminal bronchiole walls. There was also light staining underneath cells along the alveolar wall, but not of epithelial cells (Figure 3A,C). However, these cell-staining patterns were more notable in all naturally infected dogs and in dogs experimentally infected with BH401, including α-SMA-positive cells just beneath the epithelium, forming the structures “balls of black wool” previously described by Gonçalves (2003) [4] (Figure 3B,D,E). In fact, the semiquantitative analysis revealed higher α-SMA expression in naturally infected dogs and dogs experimentally infected with BH401 than in dogs experimentally infected with the BH46 strain and the controls (Scheme 1).

**Figure 4:**
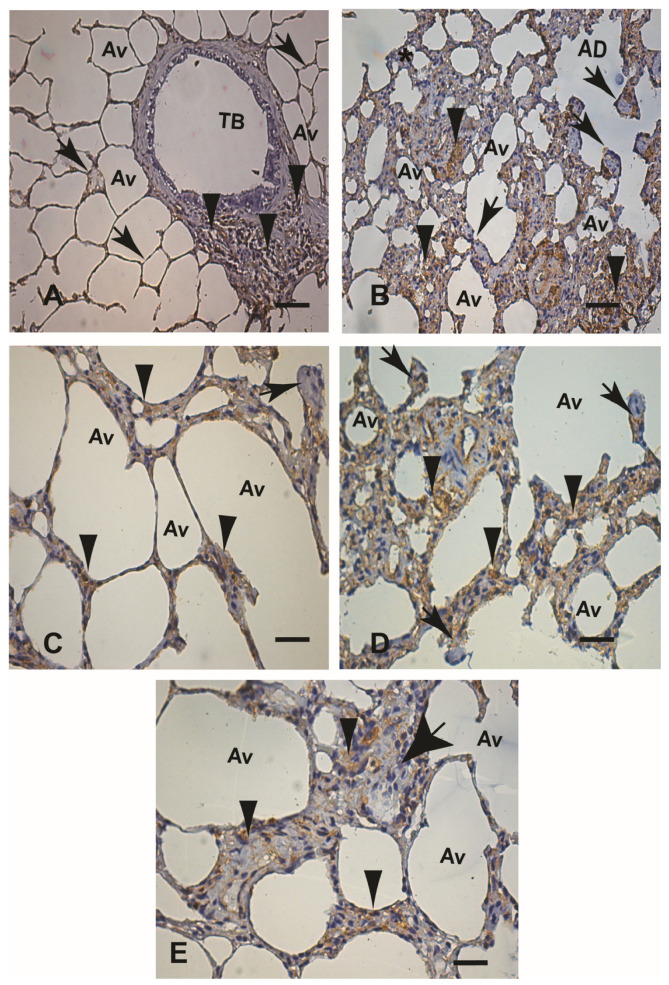
(**A**–**E**) Vimentin immunohistochemical expression in paraffin-embedded lung tissue samples from an uninfected dog (control) and an experimental dog infected with the *Leishmania infantum* BH401 strain. (**A**,**C**) Control dog: (**A**) Lower magnification showing restricted vimentin-positive cells in terminal bronchiole walls (interstitial tissue) (black arrowheads) and alveolar septa (lung parenchyma) (black arrows), 220×. (**C**) Higher magnification showing discrete vimentin positivity in the alveolar septa (black arrowheads). Note that, in the upper right corner of the figure, there is no vimentin positivity in the epithelium of the knob alveolar pulmonary structures (black arrow). (**B**–**E**) Experimentally infected dog (BH401 strain): (**B**) Lower magnification to verify vimentin-positive cells markedly following the chronic interstitial pneumonitis (black arrowheads). Note the hypertrophic and hyperplastic knob alveolar pulmonary structures, 440×. (**D**) Higher magnification showing the presence of a diffuse vimentin-positive cell exudate localized in the alveolar septa (black arrowheads). Note the hyperplastic and hypertrophy knob structures of the alveolar ducts with vimentin positivity underneath the epithelium, interstitial space (black arrowheads) 400×. (**E**) Note the thickness of the alveolar septa because of the inflammatory cell exudate with positive vimentin cell expression (black arrowheads). Note the granuloma formation in the pulmonary parenchyma (large black arrow), 440×. Immunohistochemistry streptavidin–peroxidase method with counterstaining with Harris hematoxylin. Terminal bronchiole (TB). Alveolar duct (AD). Alveolus (Av). Bars (**A**,**B**) = 20 µm. Bars (**C**–**E**) = 60 µm.

**Scheme 2:**
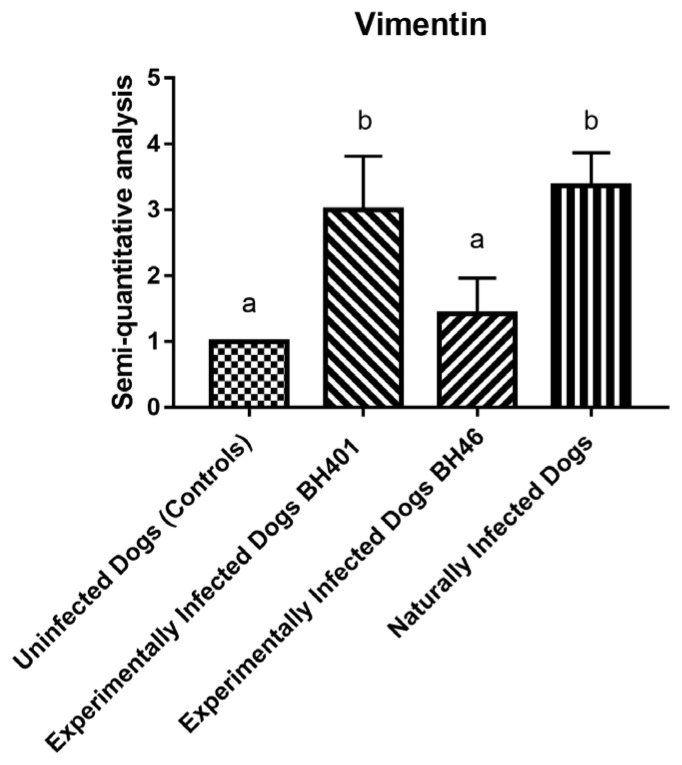
Vimentin cell expression morphometric analysis (semiquantitative study) among dog groups: dogs naturally infected with *L. infantum*, experimentally infected dogs with *Leishmania (L.) infantum* BH 401 strain, experimentally infected dogs with *Leishmania infantum* BH 46 strain, and uninfected dogs (controls). The “a” and “b” denote statistically significant difference (*p* < 0.0001).

The expression of vimentin was always diffuse and localized mainly in the lung alveolar septa (lung parenchyma) in all dogs but was quite apparent in both naturally infected dogs and dogs experimentally infected with the *L. infantum* BH401 strain. Vimentin-positive cells were easily found following the chronic cellular inflammatory exudate. However, in contrast to the restricted α-SMA cell expression, vimentin positivity was much more diffuse in the lung parenchyma, always within the lung interalveolar space (Figure 4A–E). The semiquantitative vimentin analysis revealed the same results as the α-SMA expression: higher expression in the naturally infected dogs and in the experimentally infected dogs (BH401 strain) relative to the experimentally infected dogs (BH46 strain) and controls (Scheme 2).

**Figure 5:**
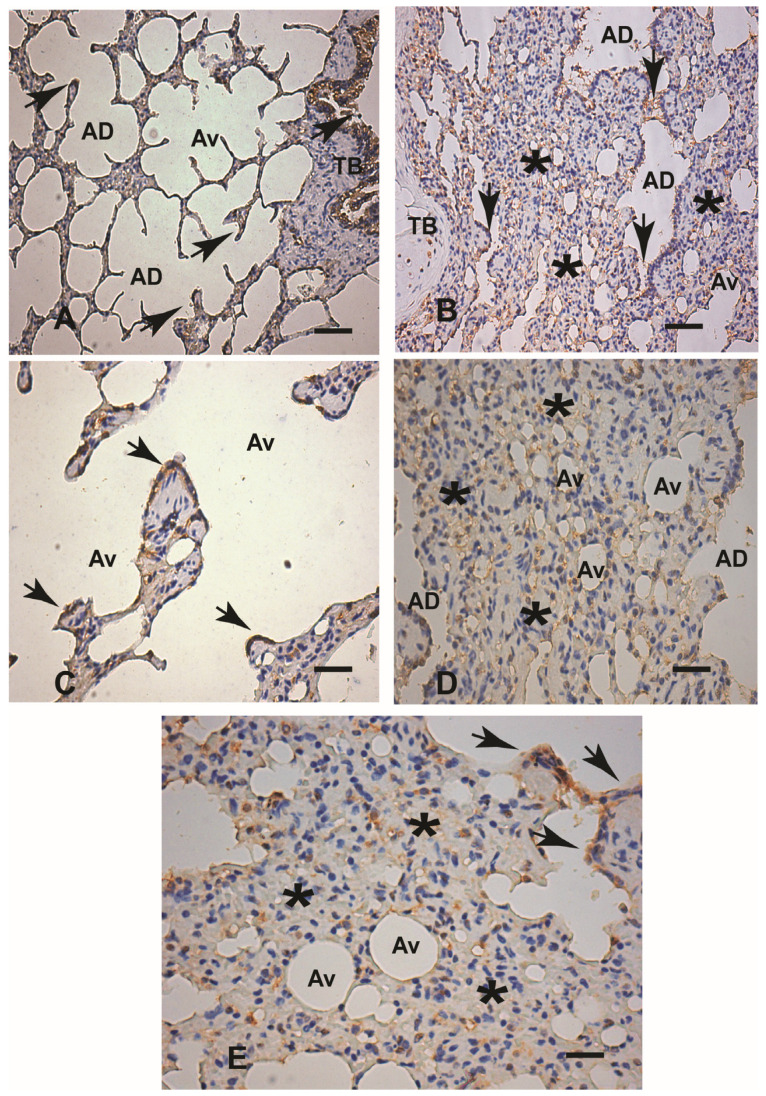
(**A**–**E**) Cytokeratin immunohistochemical expression in paraffin-embedded lung tissue samples from an uninfected dog (control dog), an experimental dog infected with the *Leishmania infantum* BH401 strain, and a dog naturally infected with *Leishmania infantum*. (**A**,**C**) Control dog: (**A**) Lower magnification showing discrete cytokeratin-positive cell expression in epithelial cells of the terminal bronchioles, alveolus ducts, and alveolus walls (black arrows), 220×. (**C**) Higher magnification showing discrete cytokeratin positivity labeling mainly in the epithelium of the arteriolar wall (black arrows), 440×. (**B**–**E**) Experimentally infected dog (BH401 strain): (**B**) Lower magnification to verify cytokeratin-positive cells markedly visible in the terminal bronchiole, alveolar ducts, and alveoli epithelium walls (black arrows). Additionally, observe the cytokeratin-positive cell following the intense chronic interstitial pneumonitis (black asterisks), 220×. (**D**,**E**) Higher magnification showing cytokeratin-positive cells in the pulmonary parenchyma (black asterisks). Note, in the right corner of figure (**E**), the presence of epithelial cytokeratin-positive cells (black arrows); 440×. Immunohistochemistry streptavidin–peroxidase method with counterstaining with Harris hematoxylin. Terminal bronchiole (TB). Alveolus duct (AD). Alveolus (Av). Bars (**A**,**B**) = 20 µm. Bars (**C**–**E**) = 60 µm.

**Scheme 3:**
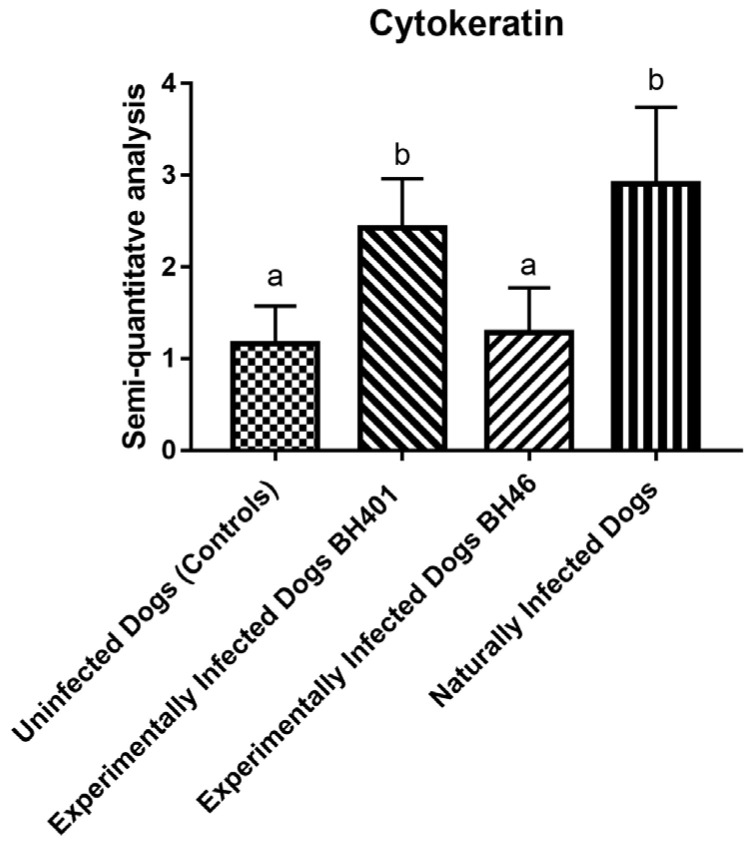
Cytokeratin cell expression morphometric analysis (semiquantitative study) among the dog groups: dogs naturally infected with *Leishmania infantum*, experimentally infected dogs with *Leishmania infantum* BH 401 strain, experimentally infected dogs with *Leishmania infantum* BH 46 strain, and uninfected dogs (controls). The “a” and “b” denote statistically significant difference (*p* < 0.0001).

Cytokeratin appeared throughout the lung parenchyma in all cases but primarily in the epithelial layers of all bronchial, terminal bronchioles, alveolar ducts, and alveoli, being always more notable in naturally infected dogs and experimentally infected dogs (BH401 strain) (Figure 5A–E). In fact, the semiquantitative analysis showed higher cytokeratin expression in both of these groups relative to the dogs infected with the BH46 strain and the uninfected controls (Scheme 3).

**Figure 6:**
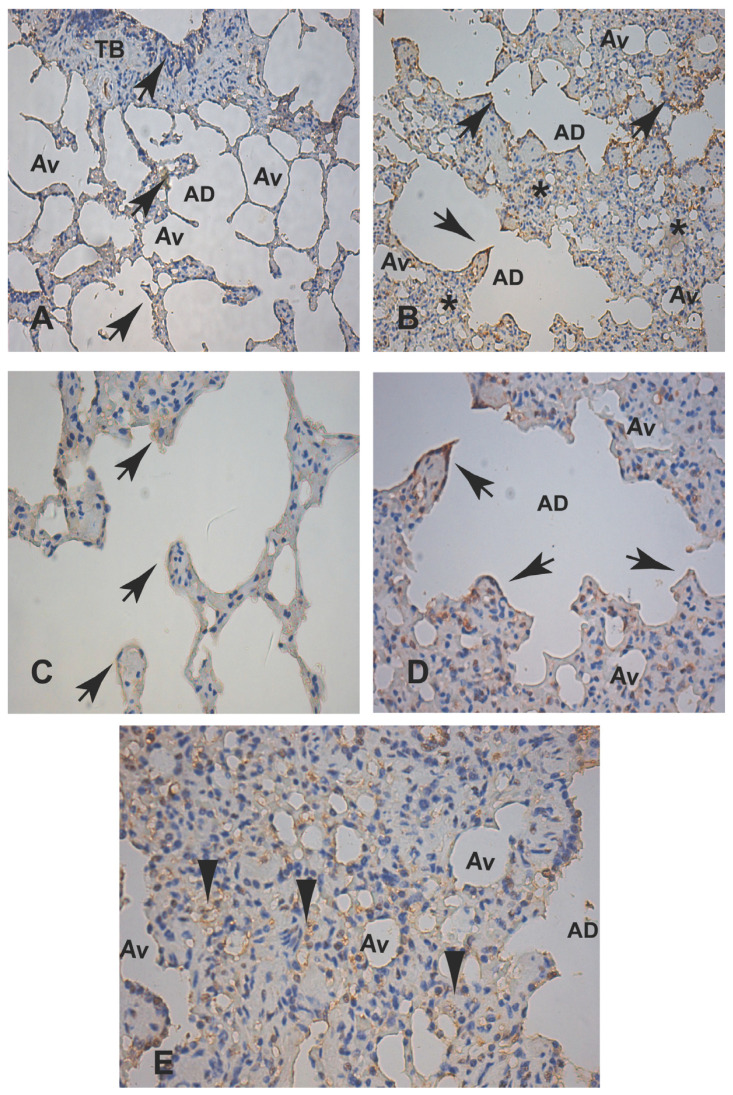
(**A**–**E**) Snail antigen homologue 1 (Snail) immunohistochemical expression in paraffin-embedded lung tissue samples from an uninfected dog (control), an experimentally infected dog with *Leishmania infantum* BH401 strain, and a naturally infected dog with *Leishmania infantum*. (**A**,**C**) Control dog: (**A**) Lower magnification showing a discrete Snail cell-labeling occurrence in the epithelium of the terminal bronchioles, alveolar ducts, and alveoli walls (black arrows), 220×. (**C**) Higher magnification showing a few Snail-positive cells (black arrows). Note the lack of inflammatory cells in the alveolar septa, 440×. (**B**,**D**) Experimentally infected dog with *Leishmania infantum* (BH401 strain): (**B**) Lower magnification showing a marked epithelium line of Snail-positive cells throughout the alveolar ducts and alveoli (black arrows). Observe an intense chronic interstitial pneumonitis characterized by the thickening of the alveolar walls with Snail-positive cells (black asterisks). (**D**) Higher magnification showing Snail-positive cells in the alveolar hyperplastic epithelium duct wall (black arrows), 440×. (**E**) Higher magnification showing Snail-positive cells in the interalveolar space following the chronic cellular exudate (black arrowheads), 440×. Immunohistochemistry streptavidin–peroxidase method with counterstaining with Harris hematoxylin. Terminal bronchiole (TB). Alveolar duct (AD). Alveolus (Av). Bars (**A**,**B**) = 20 µm. Bars (**C**–**E**) = 60 µm.

**Scheme 4:**
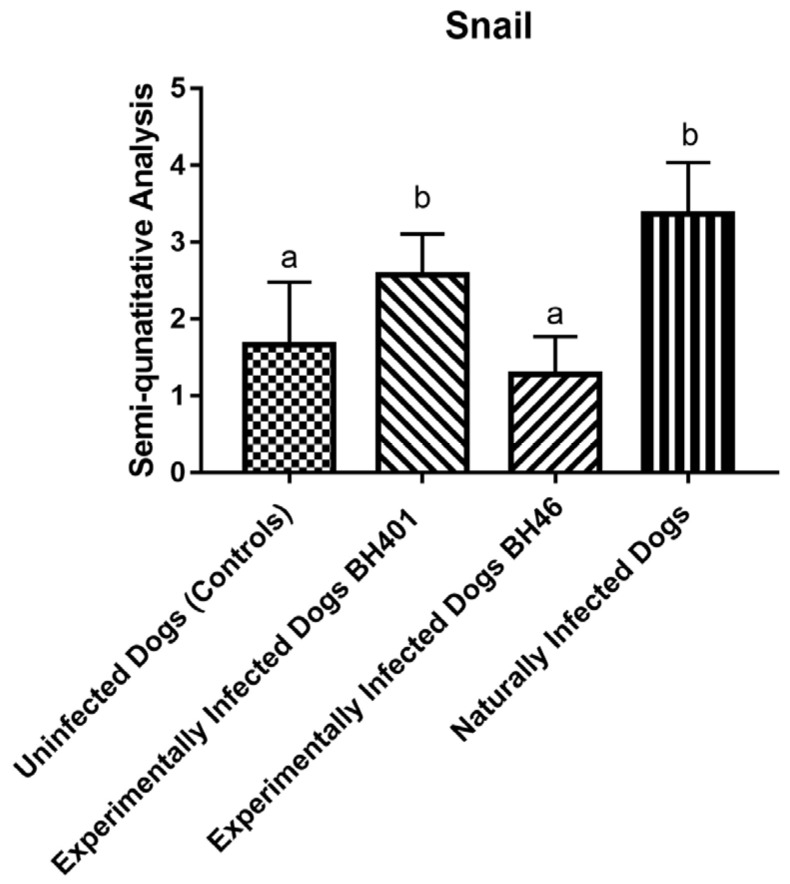
Snail antigen homologue 1 (Snail) cell expression morphometric analysis (semiquantitative study) among the dog groups: dogs naturally infected with *Leishmania infantum*, experimentally infected dogs with *Leishmania infantum* BH 401 strain, experimentally infected dogs with *Leishmania infantum* BH 46 strain, and uninfected dogs (controls). The “a” and “b” denote statistically significant difference (*p* < 0.0001).

E-cadherin cell expression was lower than the other studied molecules, but it was observed in the epithelium of the bronchial, terminal bronchioles, alveolar ducts, and alveoli walls. Under microscopic analysis, no difference in expression was found among the dog groups. On the other hand, Snail cell expression was higher in the naturally infected dogs and both groups of experimentally infected dogs (*p* < 0.0001) (Scheme 4). In fact, in both groups, in addition to the similar distribution of positive cells throughout the epithelium of the bronchial, terminal bronchioles, alveolar ducts, and alveoli walls, Snail labeling was mainly observed in pneumocytes lining the alveolar ducts and alveolar sacs (Figure 6A–D). In addition, Snail-positive cells were clearly seen in the pulmonary interstitial space following chronic pneumonitis (Figure 6E).

**Scheme 5:**
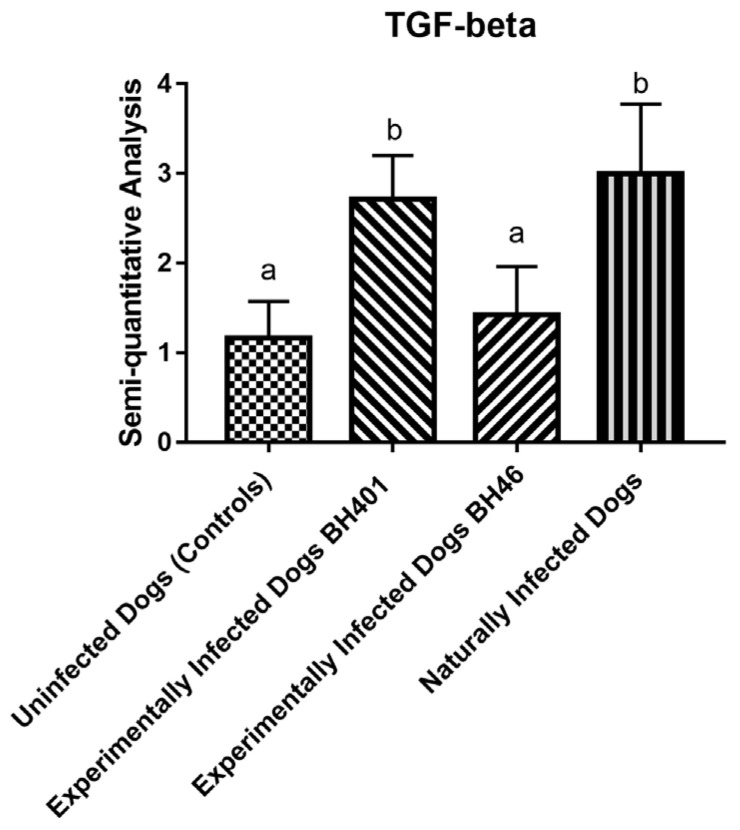
Transforming growth factor-beta (TGF-β) cytokine expression morphometric analysis (semiquantitative study) among the dog groups: dogs naturally infected with *Leishmania (L.) infantum*, experimentally infected dogs with *Leishmania infantum* BH 401 strain, experimentally infected dogs with *Leishmania (L.) infantum* BH 46 strain, and uninfected dogs (controls). The “a” and “b” denote statistically significant difference (*p* < 0.0001).

**Figure 7:**
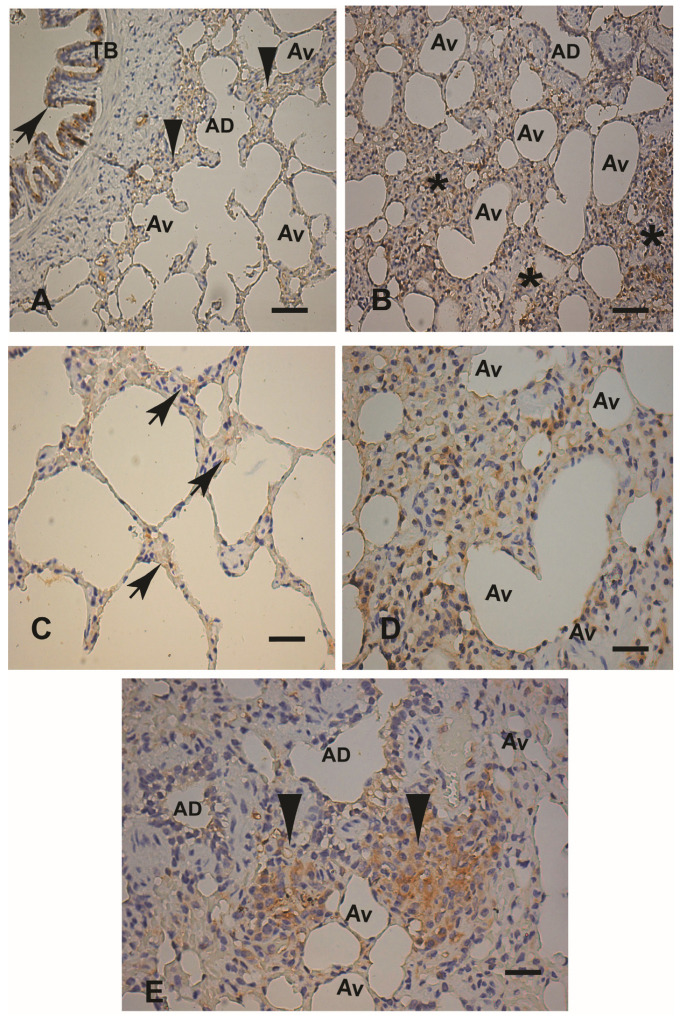
(**A**–**E**) Transforming growth factor-beta (TGF-β) cytokine immunohistochemical expression in paraffin-embedded lung tissue samples from an uninfected dog (control) and an experimentally infected dog with the *Leishmania infantum* BH401 strain. (**A**,**C**) Control dog: (**A**) Lower magnification showing a discrete TGF-β cytokine-labeling occurrence in the epithelium of the terminal bronchioles (black arrow), alveolar ducts, and alveoli walls (black arrowheads), 220×. (**C**) Higher magnification showing little TGF-β positivity in the interalveolar space (black arrows). Note the lack of inflammatory cells in the alveolar septa, 440×. (**B**,**D**) Experimentally infected dog (BH401 strain): (**B**) Lower magnification showing intense chronic pneumonitis with moderate-to-intense TGF-β positivity in the interalveolar space (black asterisks), 220×. (**D**) Higher magnification showing TGF-β positivity visible in the pulmonary parenchyma, 440×. (**E**) Higher magnification also showing the chronic cellular exudate forming two granulomas (large black arrowheads) with TGF-β positivity, 440×. Immunohistochemistry streptavidin–peroxidase method with counterstaining with Harris hematoxylin. Terminal bronchiole (TB). Alveolar duct (AD). Alveolus (Av). Bars (**A**,**B**) = 20 µm. Bars (**C**–**E**) = 60 µm.

Transforming growth factor-beta (TGF-β) cytokine was prevalent in the alveolar septa. In addition, TGF-β tissue cell expression could be seen in epithelial cells lining the bronchial, terminal bronchioles, alveolar ducts, and alveoli walls, but its expression was mainly observed diffusely in the pulmonary interstitial space mainly in the naturally infected dogs and dogs experimentally infected with the BH401 strain groups (Figure 7A–E). Indeed, after the semiquantitative analysis, the TGF-β tissue cell expression was higher in both of these groups relative to the dogs experimentally infected with the BH46 strain and the uninfected controls (Scheme 5).

## 4. Discussion

In general, intense diffuse and/or focal chronic interstitial pneumonia was the main lesion observed in naturally infected dogs and dogs experimentally infected with the BH401 strain. Dogs experimentally infected with BH46 only showed minor or absent focal chronic interstitial pneumonia with mild patchy inflammatory damage. In dogs naturally infected with *L. infantum*, chronic pneumonitis has been reported to be characterized by thickening of the alveolar septum as a consequence of an increase in cellularity (mononuclear inflammatory exudate) [3,4]. Chronic exudate of mononuclear cells composed of macrophages, plasma cells, and lymphocytes tended to form multiple foci, followed by septal thickening. However, exudate was also observed in some normal-appearing or thinner alveolar septa. As reported by Gonçalves et al. (2003) [4] and Silva et al. (2013) [11], the mononuclear infiltrate is responsible for collagen deposition, and it was confirmed by Gomori’s ammonium silver staining in lung septa throughout the hypertrophic and hyperplastic alveoli knobs (“balls of black wool”, as previously described [4]. Perivascular inflammatory infiltrate, predominantly mononuclear and peribronchiol, with similar mononuclear cellular exudate was observed in some cases.

In contrast to the dogs experimentally infected with BH401, dogs from the group experimentally infected by the BH46 strain showed no pathological changes consistent with the development of interstitial pneumonia, with no thickening of the pulmonary interalveolar septum or only minor and slight inflammatory cell exudate. This may be related to the fact that the virulence of the *L. infantum* BH401 strain was more effective in inducing lung pathology. However, it is important to say that all animals received the same care, including adequate shelter, food, and regular veterinary monitoring throughout the trials [18]. An experimental infection model for inducing chronic interstitial pneumonitis has been well described in hamsters [1]. However, there are rare reports of dogs experimentally infected with *L. infantum* and *L. donovani* describing interstitial pneumonitis [18]. In addition, there are many works with distinct experimental protocols for inducing CVL; in the literature, these manuscripts do not mention or provide extensive details about the lung anatomical pathology [19,20,21,22].

We found amastigote forms of *Leishmania* in all dogs experimentally infected with the *L. infantum* BH401 strain and in seven of eleven dogs naturally infected with *L. infantum* under H&E staining or immunohistochemistry analysis. The PCR showed positivity in the naturally infected dogs and three dogs experimentally infected with BH46. All samples from control dogs were PCR-negative. In general, in accordance with the literature, authors have reported few parasites detected in the lungs using H&E or even by immunohistochemistry of the lungs of dogs naturally infected with *L. infantum* [3,4].

Fibrosis has already been discussed in the lungs in CVL (Silva et al., 2013 [11]). Related to this behavior of fibrosis, several authors have employed biological methods to characterize this pathology. For example, in a study of idiopathic pulmonary fibrosis [23], the authors confirmed α-SMA expression in human lung myofibroblasts (HLMFs) because this protein differentiates myofibroblasts from quiescent fibroblasts. Moreover, the α-SMA expression change in HLMFs is regulated by TGFβ. Moreover, in another study of pulmonary fibrosis due to paraquat poisoning [24] in mice, these authors found that treatment with rapamycin ameliorated pathological fibrosis, including alveolar collapse and interstitial collagen deposits, where the expression of α-SMA and TGF-β was inhibited in lung tissue after this treatment. Herein, dogs experimentally infected with the *L. infantum* BH401 strain and dogs naturally infected with *L. infantum* showed higher expression of α-SMA than dogs experimentally infected with the BH46 strain and controls. These results are consistent with the higher collagen deposition verified in pulmonary septa and in the “balls of black wool” in pulmonary alveolus [4], and it suggests that myofibroblast activity contributes to pulmonary fibrosis. This finding was present in all groups, but the *Leishmania* strain BH401 induced much more severe pathology than the *Leishmania* BH46 strain. Dogs from this latter group were more similar to the control dogs.

Vimentin protein expression was more remarkable than that of α-SMA, where it was distributed throughout the lung parenchyma (but was minor in pneumocytes). There was conspicuous expression in the intra-alveolar space (lung septa) in infected dogs (except the BH46 group) when compared to controls, reinforcing the idea of mesenchymal cell activity. Cytokeratin expression followed a similar quantitative expression pattern as vimentin, and it was positive in the epithelium of terminal bronchioles, alveolar ducts, and the alveolar wall (positive pneumocytes over the structures referred to “as balls of black wool”). Again, the highest expression of cytokeratin was found in dogs experimentally infected with *L. infantum* strain BH401 and dogs naturally infected with *L. infantum*. This finding reinforces the suggestion of mesenchymal cell activity corresponding to the chronic pneumonitis fibrotic process.

TGF-β is the central cytokine in various fibrotic diseases, and it is released by cell sinusoidal capillaries, endothelial cells, CD4 T lymphocytes, monocytes, macrophages, hepatocytes, and bronchial epithelial cells [25,26,27]. We showed the expression of this cytokine in the liver related to the fibrosis process in experimentally and naturally *L. infantum*-infected dogs [15,27,28]. Here, we found high TGF-β expression in bronchial epithelial cells, as well as in areas of hypertrophy and hyperplasia of pneumocytes and in the intra-alveolar space (pulmonary interstitium). This is in agreement with previous work [24] describing ovalbumin-induced lung fibrosis in mice, with changes such as peribronchial fibrosis, fibroblast proliferation and conversion to myofibroblasts, and muscle hypertrophy. Herein, TGF-β expression followed the chronic interstitial pneumonitis fibrosis process concerning the expression data of fibrotic markers, such as α-SMA, vimentin, and cytokeratin. Thus, TGF-β was remarkably present in both the experimentally infected with *Leishmania* strain BH401 and the naturally infected dogs.

The starting point of epithelial-to-mesenchymal transition (EMT) may occur via molecules such as TGF-β; and other growth factors, such as epidermal growth factor, fibroblast growth factor, and hepatocyte growth factor. TGF-β is the most important cytokine in the restructuring of the airways [26]. Yang (2013) [27] also stated that increasing levels of TGF-β are positively correlated with Snail expression, suggesting an EMT event. Bronchial epithelial cells are able to transform into a phenotype of fibroblasts and secrete ECM compounds in response to TGF-β1 stimuli, and it is possible that epithelial cells play a role in peribronchial fibrosis and promote airway remodeling in asthma. TGF-β contributes to the development of fibroblastic foci in the bronchial area, and a number of studies have focused on the role of TGF-β1 as the key promoter of peribronchial fibrosis. Although the mechanisms of the development of pulmonary fibrosis are not entirely clear, the studies suggest that the transcription factor Snail, stimulated by the TGF-β cytokine, interferes with E-cadherin genes to inhibit this expression and trigger the EMT event. Recent studies have shown that the TGF-β/Snail/α-SMA signaling pathway plays an important role in fibrosis [29].

Snail, a zinc-finger transcription factor, is a marker of fibrotic activity via EMT. E-cadherin and cytokeratin were also investigated as epithelial cell markers. It is known that decreasing E-cadherin expression along with increasing Snail expression indicates EMT during fibrogenesis [28]. Thus, upregulation of the expression of TGF-β, Snail, vimentin, and α-SMA, in contrast to the low E-cadherin expression (no difference among groups), might suggest the activity of mesenchymal cells derived from EMT. In this work, Snail expression in the lung was higher in experimentally infected dogs with the *L. infantum* BH401 strain and naturally infected dogs, coinciding with the location of TGF-β expression (pulmonary interstitium), confirming collagen-producing cells (myofibroblasts) in the lung septa. As reported by Fang (2000) [24], epithelial and mesenchymal cells signal each other bidirectionally and dynamically, either by the release of soluble mediators via paracrine effects or by direct cellular contact. Bidirectional signaling between epithelial and mesenchymal cells involves cell–cell and cell–matrix interactions with the release of soluble mediators in a contact region known as the “epithelial–mesenchymal trophic unit.”

All infected dogs developed interstitial pneumonitis during the course of CVL, although the parasite load was variable. Two years after dogs are experimentally infected with *Leishmania*, they can develop interstitial pneumonitis, but this is dependent on the virulence of the strain of *Leishmania*. We observed fibrotic activity in the lungs of both experimental (strain BH401) and naturally infected dogs, as reflected in the expression of the fibrotic markers α-SMA, vimentin, TGF-β, and Snail. In addition, some activity of mesenchymal cells derived from the EMT during this process could be detected.

## Figures and Tables

**Table 1:** Primary antibodies used for immunohistochemistry.

Antigen *	Supplier	Clone	Isotipe	Specifity	Titration
α-Actin	Dako	1A4	mIgG2a, kappa	smooth muscle cells, myofibroblasts and myoepithelial cells	1:100
Vimentin	Dako	V9	mIgG1, kappa.	intermediate filament (IF) of the cytoskeleton of vertebrate cells	1:50
E-cadherin	AbD-Serotec	4A2c7	Mouse/IgG1, kappa	Recombinant protein corresponding to the cytoplasmic domain of human E-cadherin	1:30
Cytokeratin	Dako	AE1AE3	IgG fraction	Pan Cytokeratin (although does not detect Cytokeratin 18)	1:70
TGF-β1(v)	Santa Cruz biotechnology	sc-146	rabbit polyclonal IgG	epitope mapping at the C-terminus of TGFβ1	1:50
*Snail*	Abcam	85931	Monoclonal, IgG	This antibody gave a positive signal against Human SNAIL	1:100

* For all immunohistochemistry assays, we used goat normal sérum or PBS as a control of antibodies.

## Data Availability

Data are contained within the article.
